# Primary ectopic meningioma in the thoracic cavity: A rare case report and review of the literature

**DOI:** 10.3389/fonc.2023.1149627

**Published:** 2023-04-11

**Authors:** Xu Liu, Jiao Liu, Ting Nai, Yuxia Yang, Yuchang Hu

**Affiliations:** ^1^ Institute of Pathology, China Three Gorges University, Yichang, China; ^2^ Department of Pathology, Yichang Central People’s Hospital, Yichang, China; ^3^ Department of Obstetrics, Affiliated Renhe Hospital of China Three Gorges University, Yichang, China

**Keywords:** primary ectopic meningioma, thoracic cavity, immunohistochemistry, diagnosis, case report

## Abstract

**Background:**

Meningioma is a common type of intracranial tumor in adults. It rarely arises in the chest, with only a few case reports in the English literature. Here, we report the case of a patient with a primary ectopic meningioma (PEM) located in the thoracic cavity.

**Case presentation:**

A 55-year-old woman presented with exercise-induced asthma, chest tightness, intermittent dry cough and fatigue for several months. Computed tomography revealed the presence of a huge mass in the thoracic cavity, with no connection to the spinal canal. Lung cancer and mesothelioma were suspected, and surgery was performed. Grossly, the mass was a grayish-white solid 9.5 cm × 8.4 cm × 5.3 cm in size. The microscopic morphology of the lesion was consistent with that of typical central nervous system meningioma. The pathological subtype was transitional meningioma. The tumor cells were arranged in a fascicular, whorled, storiform and meningithelial pattern, with occasional intranuclear pseudo-inclusions and psammoma bodies. In focal areas tumor cells were considerably dense, and the cells were round or irregular in shape, with less cytoplasm, uniform nuclear chromatin, and visible nucleoli and mitoses (2/10 HPF). By immunohistochemistry, the neoplastic cells showed strong and diffuse staining with vimentin, epithelial membrane antigen and SSTR2 with variable expression of PR, ALK and S100 protein. However, the cells were negative for GFAP, SOX-10, inhibin, CD34, STAT6, smooth muscle actin, desmin, CKpan, D2-40, WT-1, CK5/6 and CD45. The highest proliferation index by Ki-67 was 15%. The abnormal expression of ALK led to the initial misdiagnosis of an inflammatory myofibroblastic tumor. After 12 months of follow-up, no disease progression was observed.

**Conclusion:**

The presence of primary ectopic meningiomas in the thoracic cavity is extremely rare, and this tumor is easily misdiagnosed clinically. Imaging is suggested to determine the location and possible differential diagnosis, while the final diagnosis should be *via* pathological examination. Immunohistochemistry is crucial for disease diagnosis. Owing to our limited knowledge of PEM, its pathogenesis and tissue of origin remain unclear. Clinicians should pay close attention to such potential patients. The present case report may provide insights into the diagnosis and therapy of patients with this tumor.

## Background

Ectopic meningioma is a tumor that occurs outside the central nervous system (CNS, including the brain and spinal canal). Its histological pattern is similar to a meningioma. It can be classified into primary or metastatic meningiomas based on their association with the CNS. Primary lesions emphasize the absence of the tumor in the CNS. Primary ectopic meningioma (PEM) has not been described in detail in the latest (fifth edition) World Health Organization (WHO) classification of CNS tumors (1). PEMs can occur in the ear, lung, nose, orbit, and neck (2–7), and PEM in the thoracic cavity is extremely rare with only a few cases in the literature (8–12). This tumor is easily misdiagnosed as hamartoma, thymoma, mesothelioma, or lung cancer. Herein, we describe the case of a 55-year-old woman who underwent surgery due to a suspected malignant tumor; however, a histopathological examination of the specimen led to the diagnosis of PEM.

## Case presentation

**Figure 1:**
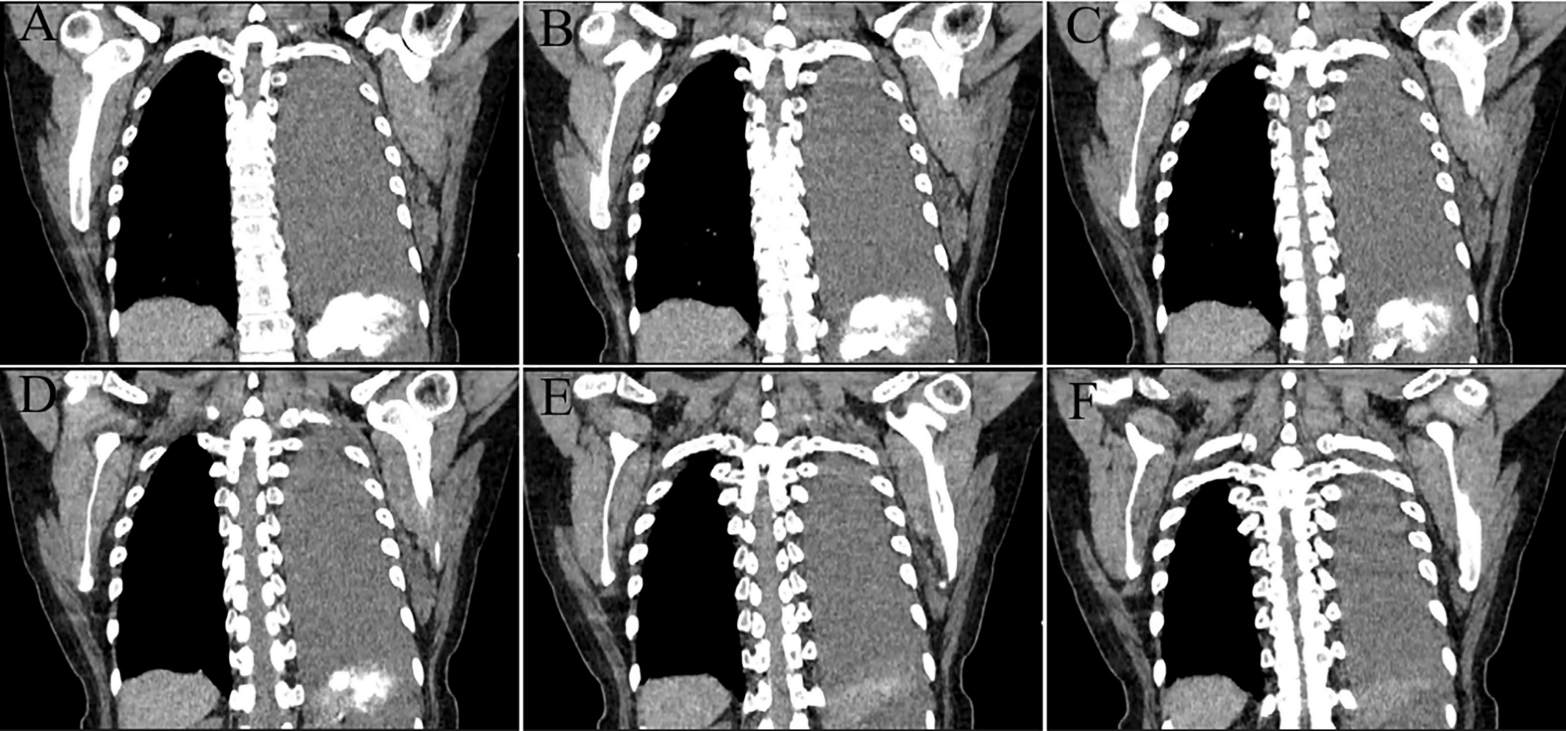
Continuous coronal computed tomography (CT) images. A mass with a high-density shadow was observed in the left thoracic cavity, with an irregular boundary and adjacent pleural thickening and adhesion. We intercepted a section of the continuous CT images from the appearance of the spinal canal to the disappearance of the tumor **(A–F)**, all of which showed no involvement of the spinal canal by the tumor.

**Figure 2:**
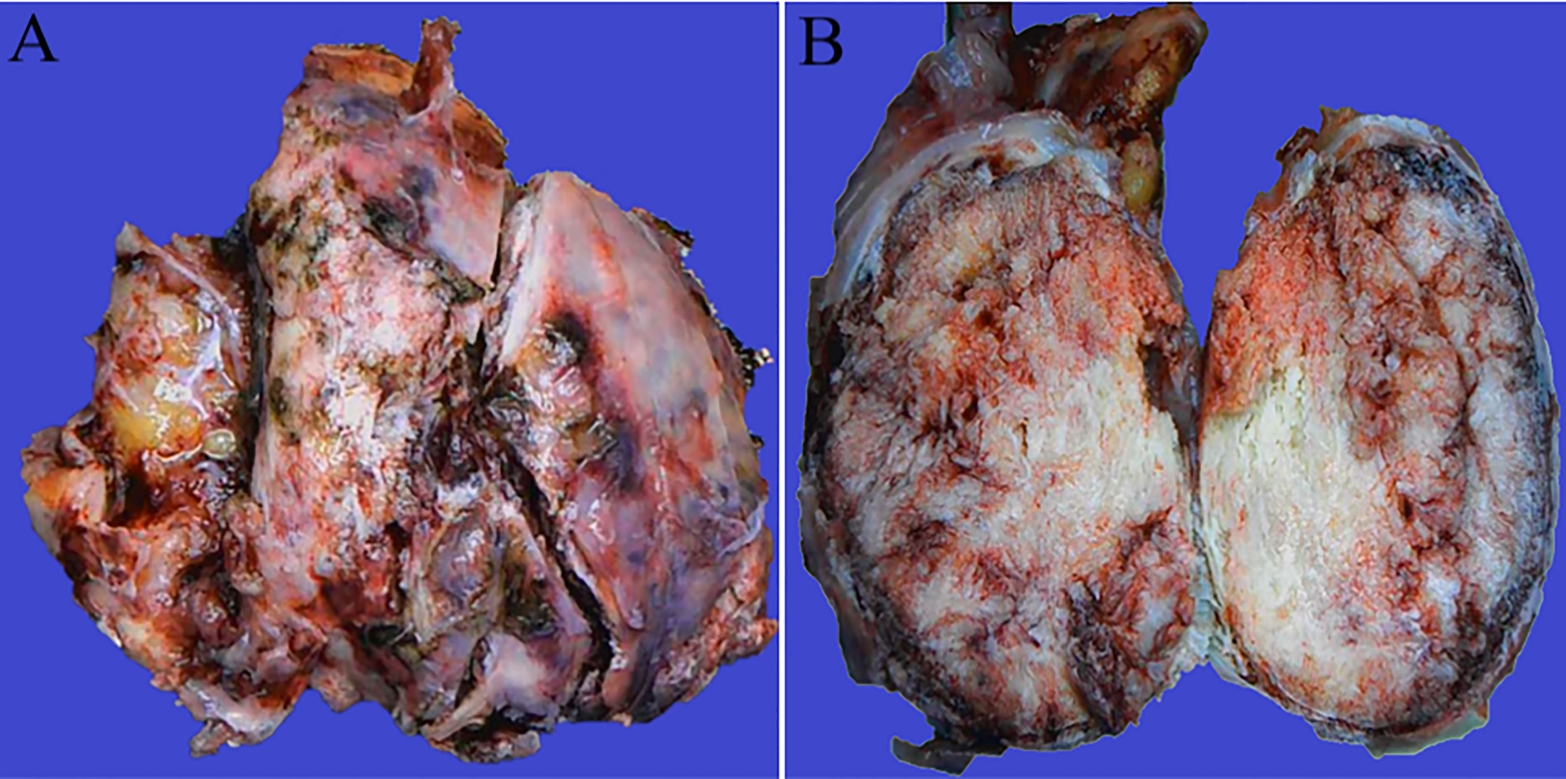
Gross pathological examination. Thoracic cavity: a grayish-white solid mass with indistinct borders **(A)**. The mass was 9.5 cm × 8.4 cm × 5.3 cm in size. Bleeding and calcification were visible in the transverse section **(B)**.

**Figure 3:**
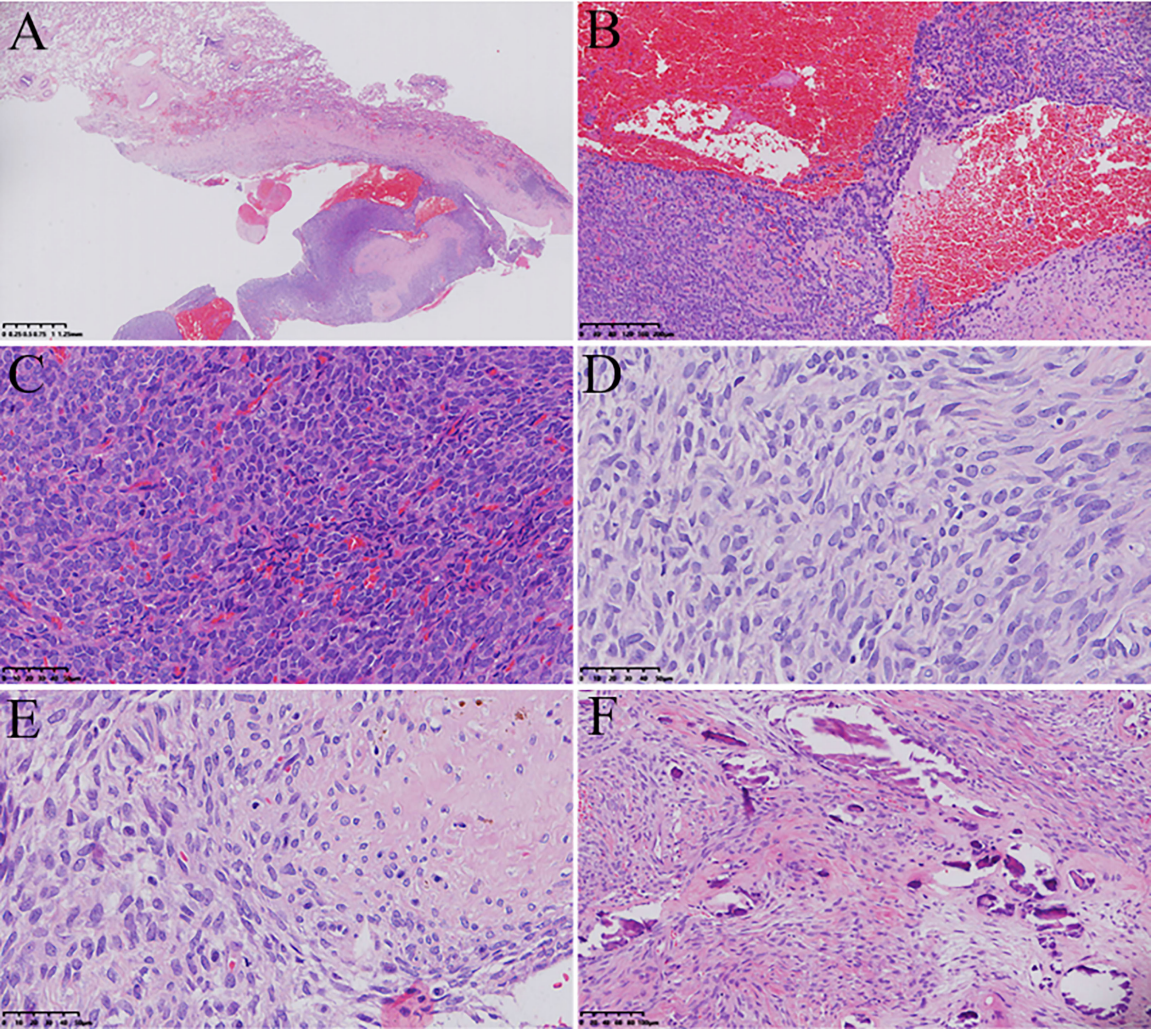
Morphology. **(A)** Scanning power showing normal alveolar structure in the upper left corner, and the tumor (underneath) is clearly separated from the lung tissue *via* pleural thickening. **(B)** A dilated hemorrhagic focus around the tumor, similar to cavernous hemangioma or hemangiopericytoma. **(C)** In the dense area of the tumor cells, the cells are round or irregular in shape, with less cytoplasm, uniform nuclear chromatin, and visible nucleoli and mitoses (2/10 HPF). **(D)** In the sparse area of the tumor cells, the cells are arranged in a fascicular, whorled, storiform and meningithelial pattern, with unclear borders between cells. These cells do not exhibit any obvious atypia, with fine chromatin, inconspicuous nucleoli, and rare mitotic figures. **(E)** Interstitial hyaline degeneration. **(F)** Presence of many psammoma bodies.

**Figure 4:**
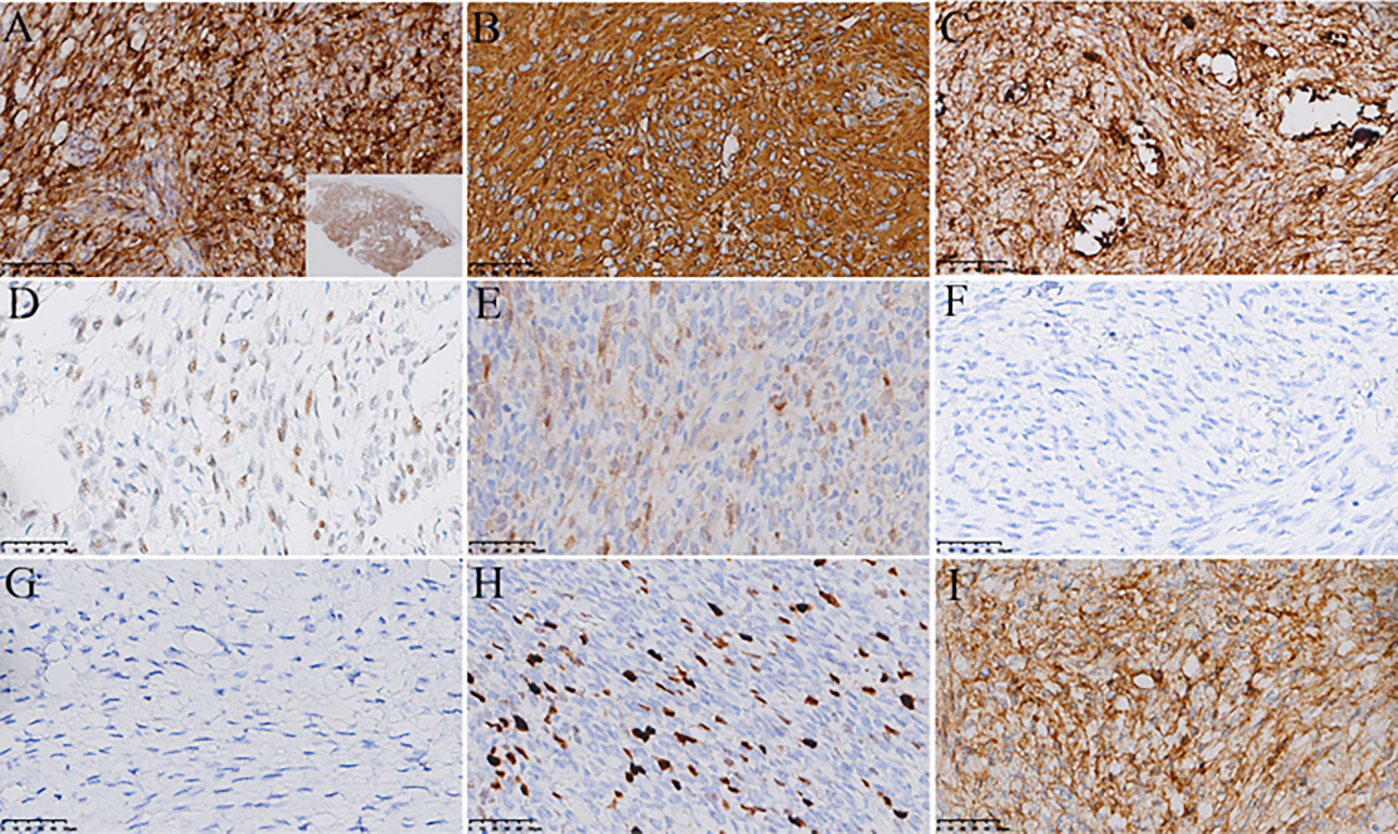
Immunohistochemistry. The tumor is diffusely and strongly positive for EMA **(A)**, scanning power is displayed in the lower right corner), vimentin **(B)** and SSTR2 **(C)**. The tumor is diffusely and weakly positive for PR **(D)**. The tumor is focally and weakly positive for S100 protein **(E)**. The tumor is negative for GFAP **(F)**, SOX-10 **(G)**, inhibin, CD34, STAT6, SMA, desmin, CKpan, D2-40, WT-1, CK5/6 and CD45. The highest proliferation index by Ki-67 is 15% **(H)**, and the tumor cells express cytoplasmic ALK **(I)**.

A 55-year-old Asian woman was admitted to our hospital with complaints of exercise-induced asthma, chest tightness, intermittent dry cough and fatigue. After admission, the symptoms could not be relieved by thoracic drainage decompression. No obvious anomalies were indicated in blood tests. She had no history of meningioma and no complaints of headaches, seizures, back pain, numbness in the hands, weakness or abnormal sensations. Chest computed tomography (CT) (**Figure 1**) revealed the presence of a large mass in the left thorax. The left lung was compressed, and a pleural effusion was observed. Subsequently, the patient underwent surgery to remove the mass. Intraoperatively, a 9-cm mass was found between the lung and the diaphragm; it was tightly adhered to the surrounding lung tissue and not attached to the spinal canal/dura. We observed that the mass had clear boundaries with the lung tissue, and the mass was not in the lung tissue. Therefore, the mass and a small amount of surrounding lung tissue were removed. Postoperative gross findings (**Figure 2**) indicated that the lesion was a grayish-white solid mass with indistinct borders. The boundary of the mass was blurred at low magnification and involved the lung surface pleura (**Figure 3A**); however, the alveolar structures were normal, and the histomorphology of the mass was distinct from that of alveolar structures. The lesion was surrounded by dilated hemorrhagic areas resembling cavernous hemangioma or hemangiopericytoma (**Figure 3B**). Under high magnification, diffuse tumor cells were observed. In the dense area of the tumor cells, the cells were round or irregular in shape, with less cytoplasm, uniform nuclear chromatin, and visible nucleoli and mitoses (2/10 HPF) (**Figure 3C**). In the sparse area of the tumor cells, the cells were arranged in a fascicular, whorled, storiform and meningithelial pattern (**Figure 3D**), with occasional intranuclear pseudo-inclusions or interstitial hyaline degeneration (**Figure 3E**). The tumor cells had abundant eosinophilic cytoplasm. Further, the nucleoli and nuclear divisions were barely visible. Lots of psammoma bodies were observed in the tumor tissue (**Figure 3F**), but no obvious inflammatory infiltration or necrosis were observed. Immunohistochemistry (**Figure 4**) revealed that the tumor cells expressed vimentin, epithelial membrane antigen (EMA), SSTR2, PR, S100 protein and ALK (Clone D5F3). ALK expression appeared in the cytoplasm. In addition, the highest proliferation index by Ki-67 was 15%. On the other hand, the cells were negative for GFAP, SOX-10, inhibin, CD34, STAT6, smooth muscle actin (SMA), desmin, CKpan, D2-40, WT-1, CK5/6 and CD45. Based on these findings, the patient was diagnosed with PEM in the left thoracic cavity and the pathological morphology was classified as transitional (mixed meningothelial and fibroblastic subtypes) WHO grade 1 meningioma. She underwent a CT of the head after diagnosis; no CNS tumor was observed. No further treatments were planned, and the patient was advised to visit the hospital regularly. During the 12-month follow-up period, the patient underwent CT in other hospitals; no recurrence or metastasis was observed.

## Discussion

Meningiomas are relatively common intracranial tumors and account for approximately 37.6% of all primary CNS tumors. However, PEM is very rare, accounting for approximately 0.4%-2% of the meningiomas reported in the literature (10, 13). In patients with PEM, intracranial and intraspinal meningiomas are absent. Therefore, the diagnosis of PEM is more rigorous, and the actual incidence may be lower. In the current case, the patient had no history of meningioma and a comprehensive CT examination eliminated CNS meningiomas. The histologic origin of PEM is unknown, and the most common hypothesis is that it originates from ectopic or migrating arachnoid cells. It has also been suggested that PEM has a different histological origin than CNS meningiomas and more likely originates from perineural cells or pluripotent mesenchymal cells (14, 15). At present, PEM mostly arises in the lungs, mediastinum, ear, nasal cavity, and neck. The age of onset can range from children to the elderly; however, PEM is more common in middle-aged and elderly women.

The clinical manifestations of PEMs are closely related to their site of occurrence, and tumors in different sites may have different clinical symptoms. If a meningioma is present in the chest, the symptoms may be dysphagia, chest tightness, asthma, intermittent dry cough, and fatigue; if a meningioma is present in the lung, the symptoms may be paroxysmal cough and expectoration; if a meningioma is present in the nose, the symptoms may be bleeding and nasal congestion; if a meningioma is present in the ear, the symptoms may be pain, swelling, and hearing loss; and if a meningioma is present in the neck, there may be no obvious symptoms. All these symptoms indicate PEM. Blood tests do not indicate any specific parameters, and imaging detects space occupying lesion. The tumor usually presents as a low-density soft tissue shadow with irregular borders; however, if there is significant calcification within the tumor, a high-density shadow may appear.

PEM is rare, and can therefore be misdiagnosed as other diseases. In our case, PEM was misdiagnosed as mesothelioma and lung cancer, which was finally clarified *via* pathological examination. The main clues for diagnosis are that the tumor cells are arranged in a fascicular, whorled, storiform and meningithelial pattern, with unclear borders between cells; these cells do not exhibit any obvious atypia, with fine chromatin, inconspicuous nucleoli, and rare mitotic figures. Meanwhile, occasional intranuclear pseudo-inclusions or psammoma bodies are observed. Three-grade classification of meningiomas with the accordingly growing risk of aggressive behavior of the tumor has been proposed by the WHO, and almost 90% are WHO grade 1 (benign) meningiomas. There are nine subtypes of WHO Grade 1 meningiomas, and ours is the transitional subtype, which is a mixture of meningothelial and fibroblastic subtypes. Immunohistochemistry plays an important role in PEM diagnosis. In general, most tumor cells express vimentin, EMA, PR, and SSTR2 and some express S100 protein. However, they do not express GFAP, SOX-10, inhibin, CD34, SMA, desmin, CKpan and CK5/6. In our case, the tumor cells abnormally expressed ALK, and the positive signals were distributed in the cytoplasm, suggesting ALK gene fusion. Unfortunately, we did not conduct fluorescence *in situ* hybridization or next-generation sequencing. However, the relationship between ALK and meningioma remains unclear and needs to be further investigated. Positive ALK expression increases the difficulty of differentiation from an inflammatory myofibroblastic tumor (IMT). The highest proliferation index by Ki-67 in our case was 15%, suggesting that the patient should be closely followed up. Many studies have shown that patients with higher Ki-67 (cutoff value >4%) should be regularly monitored to prevent a recurrence (16). The level of Ki-67 is positively correlated with tumor grade, whereas PR expression is negatively correlated with tumor grade (17, 18). Allelic loss of 22q12.2 (the region encoding the NF2 gene) is the most commonly reported genetic anomaly in meningiomas (19, 20). Other genetic anomalies, include AKT1, TRAF7, SMO, PIK3CA, KLF4, SMARCE1, BAP1, H3K27me3 and CDKN2A/B. Although the diagnostic criteria are clear, PEM diagnosis remains challenging due to its rarity and uncertain location. Some researchers suggest the use of fine-needle aspiration cytology and immunocytochemistry for the definitive diagnosis of PEM (21, 22).

Based on the location of PEM, it is necessary to distinguish it from tumors with similar morphology. PEM occurring in the thoracic cavity should be differentiated from sarcomatoid carcinoma, solitary fibrous tumor, IMT, mesothelioma, and thymoma. The differentiation between our case and IMT mainly include that IMT is more common in young people, with microscopic lymphocyte infiltration, and immunohistochemical expression of ALK, SMA, Desmin and CKpan, but no expression of EMA, SSTR2 and PR. More importantly, it should be differentiated from primary pulmonary meningioma (PPM). The clinical symptoms of PEM and PPM are similar; however, the main lesion of PPM is located in the lung. Although PPM could break through the lung membrane growth, intraoperative findings and postoperative pathology in our case revealed that there was a distinct pleural thickening separation between the tumor and the lung or the alveoli. Additionally, the main lesion was in the thoracic cavity; therefore, our case was not originating from the lung. There are many recent reports about PPM; this may be owing to the deepened understanding of minute pulmonary meningothelial-like nodules (MPMNs). At present, most scholars believe that MPMNs are reactive proliferative lesions of pulmonary meningeal epithelial cells, often with multiple nodules that are usually <0.5 cm in diameter. On the other hand, PPMs are neoplasms with obvious genetic anomalies and a relatively larger diameter (23, 24). Pathologically, MPMNs are typically characterized by proliferative lung mesenchymal cells with clear borders, usually without an envelope, and a homogeneous round or ovoid swirling cell arrangement that can grow along the alveolar septa or around blood vessels; these cells do not exhibit obvious atypia and resemble meningeal epithelial cells, with fine chromatin, inconspicuous nucleoli, and rare mitotic figures (25, 26). PPMs usually form encapsulated masses with solid, nested clusters of tumor cells, usually without an alveolar lumen and residual alveolar epithelial cells (24).

Surgery is the main treatment for PEM; extended excision is performed to ensure that there is no residual lesion. The most important factor in determining prognosis is the completeness of surgical excision (27). Furthermore, patients with atypical and malignant ectopic meningiomas (WHO grade 2-3) have a higher recurrence and mortality rate. Our patient underwent extended resection of the tumor, and she had a good prognosis during the 12-month follow-up period. Further, the patient thought the treatment plan was appropriate and was satisfied with the treatment effect.

## Conclusion

Here, we reported a rare case of PEM in the thoracic cavity. Disease diagnosis must exclude CNS meningiomas. The clinical manifestations of PEMs are not specific and vary according to their location. Laboratory tests are also unremarkable. Imaging is helpful in disease diagnosis, and the final diagnosis should be confirmed *via* pathology. Positive ALK expression increases the difficulty of differentiation from IMT. Surgery is the best treatment option. Patients with complete tumor resection and WHO grade 1 meningioma have a relatively good prognosis. We believe that a detailed clinical history, comprehensive imaging, typical pathological morphology, and appropriate immunohistochemistry are required for definitive diagnosis and treatment. We hope that more researchers pay close attention to PEM and further elaborate on the clinicopathological characteristics of this disease *via* large-sample data and study of molecular mechanisms.

## Data availability statement

The original contributions presented in the study are included in the article/supplementary material. Further inquiries can be directed to the corresponding author.

## Ethics statement

The studies involving human participants were reviewed and approved by The Ethics Committee of Yichang Central People’s Hospital (China). The patients/participants provided their written informed consent to participate in this study. Written informed consent was obtained from the individual(s) for the publication of any potentially identifiable images or data included in this article.

## Author contributions

XL and JL participated in the study design. XL wrote the manuscript. TN and YY participated in the literature search for the study and collected the images. YH revised the manuscript. All authors contributed to the article and approved the submitted version.
